# Activity and Safety of Inhaled Itraconazole Nanosuspension in a Model Pulmonary *Aspergillus fumigatus* Infection in Inoculated Young Quails

**DOI:** 10.1007/s11046-015-9885-2

**Published:** 2015-03-20

**Authors:** Piotr Wlaź, Sebastian Knaga, Kornel Kasperek, Aleksandra Wlaź, Ewa Poleszak, Grażyna Jeżewska-Witkowska, Stanisław Winiarczyk, Elżbieta Wyska, Thorsten Heinekamp, Chris Rundfeldt

**Affiliations:** 1 Department of Animal Physiology, Institute of Biology and Biochemistry, Faculty of Biology and Biotechnology, Maria Curie-Skłodowska University, Akademicka 19, PL-20033 Lublin, Poland; 2 Department of Biological Bases of Animal Production, University of Life Sciences, Lublin, Poland; 3 Department of Pathophysiology, Medical University of Lublin, Lublin, Poland; 4 Department of Applied Pharmacy, Medical University of Lublin, Lublin, Poland; 5 Department of Epizootiology and Clinic of Infectious Diseases, Faculty of Veterinary Medicine, University of Life Sciences, Lublin, Poland; 6 Department of Pharmacokinetics and Physical Pharmacy, Collegium Medicum, Jagiellonian University, Kraków, Poland; 7 Department of Molecular and Applied Microbiology, Leibniz Institute for Natural Product Research and Infection Biology, Jena, Germany; 8 Drug-Consulting Network, Melanchthonstr. 11, D-01640 Coswig, Germany

**Keywords:** Nanosuspension, Inhalation, Experimental *Aspergillus fumigatus* infection, Antifungal treatment, Avian

## Abstract

Pulmonary aspergillosis is frequently reported in parrots, falcons, and other birds held in captivity. Inhalation is the main route of infection for *Aspergillus fumigatus*, resulting in both acute and chronic disease conditions. Itraconazole (ITRA) is an antifungal commonly used in birds, but its administration requires repeated oral dosing, and the safety margin is narrow. To investigate the efficacy of inhaled ITRA, six groups of ten young quails (*Coturnix japonica*) were inoculated intratracheally with 5 × 10^6^ spores (3 groups) or 5 × 10^7^ spores (3 groups). Animals were exposed to nebulized ITRA nanosuspension as 10 % suspension or 4 % suspension, once daily for 30 min, starting 2 h after inoculation for 6 days. Control groups were exposed to nebulized saline for the same period of time. Survival and clinical scores were evaluated, and animals were subjected to gross pathology. In control animals, aspergillosis resulted in systemic disease without pulmonary or air sac granulomas. Animals died from multiple organ failure. Inhalation of 10 % ITRA nanosuspension blocked lethality and prevented disease-related symptoms in the quails exposed to the low dose of spores, while the disease course in quails inoculated with the high-spore dose was retarded. Inhalation of 4 % ITRA nanosuspension was less effective. Both inhalations were well tolerated, and gross pathology did not reveal signs of local toxicity. The data indicate that inhaled administration of 10 % ITRA nanosuspension is capable of alleviating an acute *A. fumigatus* infection in quails. A lower ITRA concentration may be only active in chronic pulmonary aspergillosis.

## Introduction

Aspergillosis is the most common fungal disease in birds and is in most cases caused by *Aspergillus fumigatus* although other *Aspergillus* spp. are occasionally involved [2, 7]. Infections caused by *A. fumigatus* have been reported in falcons and other birds of prey [8], parrots [23], and penguins [25] held in captivity. Pulmonary aspergillosis is frequently found also in wildlife centers, largely impairing the rehabilitation success of birds [1, 25]. Inhalation is considered the main route of infection for *Aspergillus* species in birds [18]. The conidia of *A. fumigatus* are much smaller than those of other species of *Aspergillus*. They are too small to be trapped in the nasal cavity; this may explain why *A. fumigatus* is the predominant species of airborne fungal infections in birds [12, 19].

Treatment of this disease is probably one of the most challenging tasks of avian practitioners [13]. In recent years, the azole derivative voriconazole has been introduced in the treatment of avian aspergillosis [10, 15, 22]. However, as with other azole derivatives, the oral treatment is hampered by the fact that the safety margin is narrow and toxicity induced by systemic administration of azoles is well known, requiring the careful balancing of administered dose and induced toxicity [4, 24]. Oral administration requires repeated fixation, which imposes stress on the birds. Therefore, alternative treatment regimens are of high interest.

An azole formulation that can be nebulized to administer the drug in an aerosol by inhalation should solve many of these issues. Recently, we have described a nanoparticulate suspension of itraconazole (ITRA), which can be nebulized using standard pressurized air-driven devices [20]. The droplet size was independent of the technology in the respirable size range of 5 µm. The suspension was proven to be stable upon storage and could be manufactured with reproducible quality. The inhaled administration in rats and quails resulted in high local drug concentration, indicating good deposition in the respiratory tract, while systemic exposure was reduced, opening up the chance to reduce the risk for systemic toxicity [20, 21]. To evaluate whether the inhaled administration of ITRA nanosuspension can alleviate the course of experimentally induced aspergillosis, an intratracheal challenge model in quails was selected, which had been used previously to evaluate the antifungal activity of orally administered voriconazole [24].

## Materials and Methods

### Animals

Clinically healthy quails (*Coturnix japonica*) of both sexes, aged 10 days (25–30 g body weight), were used for the experiments. Quails of this age were selected, since juvenile animals are more susceptible to *A. fumigatus* infection than adult animals [5, 24]. The animals were obtained from Didactic Experimental Station of the University of Life Sciences in Lublin (Poland) and were housed in groups of 20 in collective cages at a controlled temperature (26–28 °C) and humidity (50–60 %) under continuous lighting (natural and artificial). They were fed ad libitum commercial diet (DKA-Prestarter, Agropol Sp.J., Motycz, Poland). Birds had free access to fresh water during the experiment. The experimental protocols were approved by the Ethical Committee of the Medical University in Lublin (license number 28/2013). All procedures involving animals and their care were conducted in accordance with the European Communities Council Directive of November 24, 1986 (86/609/EEC) and Polish legislation acts concerning animal experimentation.

### Inoculum

*Aspergillus fumigatus* ATCC 46645 wild-type strain was used for the experiments. This strain was selected since it is a standardized strain with reproducible characteristics and known sensitivity to ITRA. Depending on the test system used, the minimal inhibitory concentration (MIC) of this strain was shown to be in the range of 0.125–0.5 µg/ml [26]. *A. fumigatus* ATCC 46645 was grown on malt extract agar. Five-day-old cultures were washed with 5 ml 0.01 % polysorbate 80 in phosphate-buffered saline (PBS) to harvest conidia. The spore suspension was filtered through a cell strainer (40 µm pore size; BD Biosciences, Germany) and washed three times in 0.01 % polysorbate 80 in PBS. The number of conidia was determined using a CASY cell counter (model TT; Roche Innovatis AG, Germany), and the suspension was adjusted to a concentration of 10^9^ conidia/ml PBS.

### ITRA Nanosuspension

The nanoparticulate ITRA suspension was prepared as described previously [20]. Briefly, a wet-milling process using a pearl mill (VMA Getzmann, Reichshof, Germany) with organic grinding beads was used. Microcrystalline pure ITRA drug powder (Matrix Laboratories Ltd, Secunderabad, India) was suspended in distilled water at a concentration of 10 % by weight, with the addition of polysorbate 80 at 1.4 % using a high shear mixer (Ultra-Turrax homogenizer, IKA-Werke GmbH & Co, Staufen, Germany). The suspension was milled for 4 h. The resulting suspension had a mean particle size of 180–230 nm with a narrow particle size distribution and was found to be stable upon storage at 8 °C for at least 9 months. To generate a suspension containing 4 % ITRA, 10 ml of the 10 % suspension was mixed with 15 ml of a 1.4 % polysorbate 80 solution in water using a magnetic stirrer. Upon release testing, the particle size distribution of ITRA particles was found to be in the range of 180–230 nm with narrow particle size distribution, and the suspension could be nebulized with droplet size in the range of less than 5 µm using Pariboy SX with PARI LC Sprint nebulizer equipped with red insert for fine droplets (Pari GmbH, Starnberg, Germany).

### Statistical Analysis

The survival data are displayed as Kaplan–Meier survival plot. The statistical analysis of survival differences between groups was done with a log-rank test. All infected birds were included. Cumulative disease scores were plotted as group sum data. The statistical analysis of the disease scores was based on the area under the disease score plot for the treatment compared to vehicle treatment, using the Chi-square test with α-adjustment for multiple comparisons.

### Pilot Experiment to Identify the Optimal Inoculation Dose

To identify the optimal inoculation dose, three groups of 10 male 10-day-old quails were administered 5 × 10^5^, 5 × 10^6^, or 5 × 10^7^ conidia intratracheally. For instillation, each animal was taken in one hand. The beak was opened, and using a small animal 26G intravenous catheter with the stylet removed attached to a 1-ml syringe filled with 0.1 ml suspension, the suspension was administered directly and deep into the trachea as bolus. Animals were held for 10 s after the instillation with the head up to prevent reflow of the suspension. Thereafter animals were returned to the observation cages. Ten uninfected control birds were intratracheally instilled with 0.1 ml of polysorbate 80 (0.05 % vol/vol) in saline.

Following administration, the birds were observed once daily for 9 days (day of instillation = day 1) for behavioral signs of disease and for mortality. The study duration was selected based on the disease course, resulting in spontaneous recovery of surviving animals within less than 2 weeks and no deaths after day 7 [24]. A composite disease score was constructed from the following observation parameters: general signs of disease/discomfort (0 absent, 1 equivocal, 2 present), somnolence (0 absent, 1 equivocal, 2 present), respiratory impairment (0 absent, 1 open beak, 2 open beak + tail co-movement), survival (0 alive, 8 death). Each parameter was determined once daily. In the case of death, the value of 8 was carried forward. In addition to the disease score, the mortality was evaluated separately. The body weight was recorded on days 1, 4, 6, and 8 during the study. Gross pathology was conducted on each animal which died and on the remaining animals on day 9.

**Fig. 1:**
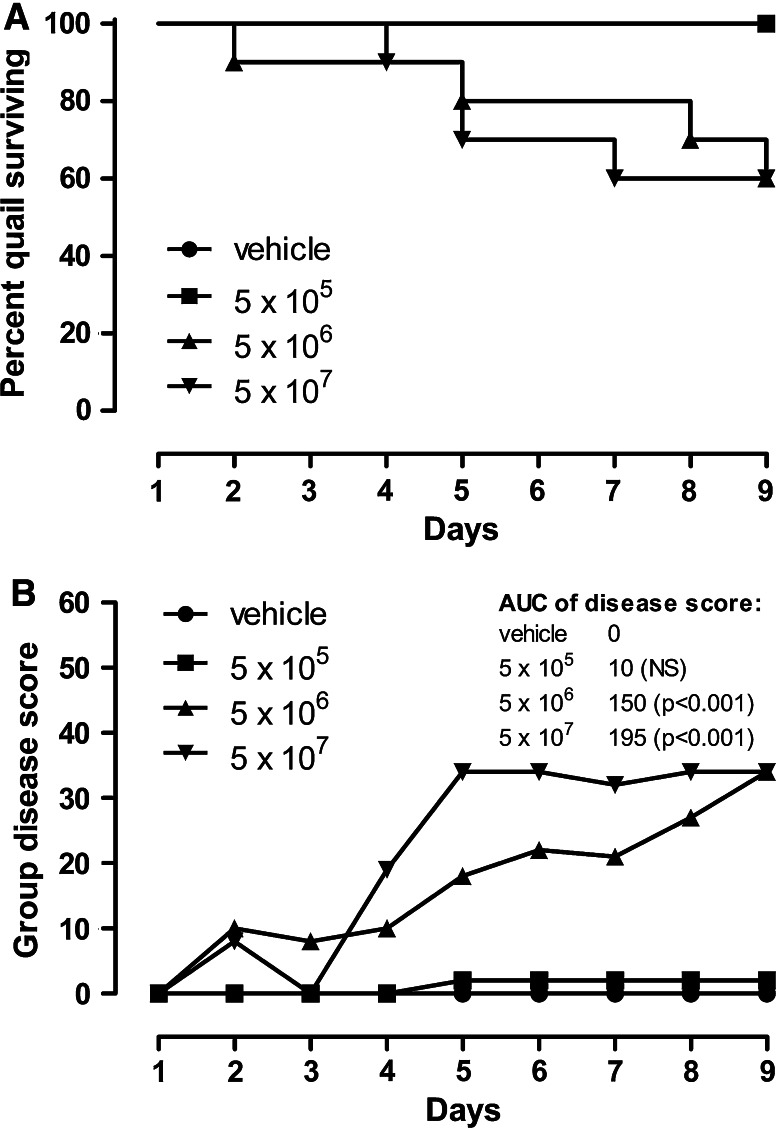
Kaplan–Meier survival curve (**a**) and cumulative disease score (**b**) of groups of quails inoculated intratracheally with 5 × 10^5^, 5 × 10^6^, or 5 × 10^7^ spores of *A. fumigatus* (*n* = 10/group). The log-rank test revealed that the inoculation with 5 × 10^6^ or 5 × 10^7^ spores significantly reduced survival (*p* = 0.02). A cumulative disease score was generated by adding up all scores of all animals on the respective day. For statistical analysis of the resulting group disease score, the area under the score curve (AUC) was calculated and compared using the Chi-square test with α-adjustment for multiple comparisons (inserted table)

The inoculation with 5 × 10^5^ conidia did not result in a clinically manifest disease. With the exception of one animal with possible mild symptoms of disease, starting on day 5, no symptoms were recorded. In contrast, the administration of either 5 × 10^6^ or 5 × 10^7^ conidia resulted in a fulminant disease development with first signs of disease becoming visible on the day following inoculation. The disease development was somewhat protracted with 5 × 10^6^ conidia, but the difference was small. In both groups, the lethality reached 4 of 10 animals on day 9 (Fig. 1a, b). The *A. fumigatus* inoculation had no consistent effect on the group mean body weight development. Gross pathology revealed unspecific signs of cardiovascular failure including lung hyperemia, but no fungal granulomas and no macroscopically visible fungal growth were seen on the air sac surfaces, in line with the acute course of the disease (data not shown).

### Treatment Regimens

Due to the similarity in disease development following inoculation of 5 × 10^6^ and 5 × 10^7^ conidia, it was decided to evaluate the pharmacological effect of inhaled ITRA nanosuspension in both quails inoculated with 5 × 10^6^ conidia (low inoculation groups) and 5 × 10^7^ conidia (high inoculation groups). Two groups of 30 quails each were inoculated either with 5 × 10^6^ or 5 × 10^7^ conidia. The animals of each group were randomized to three treatment groups of ten quails each, to be treated with 10 % suspension, 4 % suspension, or vehicle. Starting 2 h after the inoculation, the animals were put in an inhalation cage and were exposed to nebulized ITRA nanosuspension for 30 min, as described previously [21]. In brief, a polycarbonate Eurostandard type III rodent cage (bottom area 390 × 230 mm, height 150 mm, total volume ∼13.5 l) was equipped with a tightly sealing custom-made acrylic glass cover with an inlet in the left front corner (18 mm diameter) and a tubing outlet in the right rear corner (18 mm diameter) to allow for a diagonal airflow through the chamber. The inlet was connected directly with a short section of silicone tubing (25 cm long, 18 mm diameter) to a pressurized air nebulizer (Pari Boy SX with PARI LC Sprint nebulizer equipped with red insert for fine droplets (Pari GmbH, Starnberg, Germany). To prevent quail from hiding in corners or blocking the inlet or outlet to enable a homogenous drug inhalation, the cage was equipped with a circular plastic mesh sized to block the corners and to protect the inlet and outlet. All 10 quails of each group were placed in the cage for simultaneous exposure. The inhalation procedure was conducted once daily in the late morning for six consecutive days. The animals were observed for the development of the disease using the composite disease score and for mortality once daily starting the day of inhalation. In addition, the body weight development was recorded on days 1, 4, and 8. Gross pathology was conducted on each animal which died and on the remaining animals on day 8.

## Results

ITRA nanosuspension could be easily nebulized using Pariboy SX with PARI LC Sprint nebulizer equipped with red insert for fine droplets (Pari GmbH, Starnberg, Germany). Within 30 min of nebulization, a volume of about 4.5 ml of suspension could be successfully nebulized. Based on this nebulization capacity and the air flow generated by Pariboy SX, the concentration of ITRA per ml air amounted to 3 µg/ml air for the 10 % suspension. The administered dose cannot be easily calculated from these values, since the respiratory frequency and the respiratory volume of 10-day-old quails are not known. The inhalation was well tolerated, and no visible signs of adverse effects were observed in the quails exposed to nebulized ITRA from a 4 or 10 % nanosuspension.

**Fig. 2:**
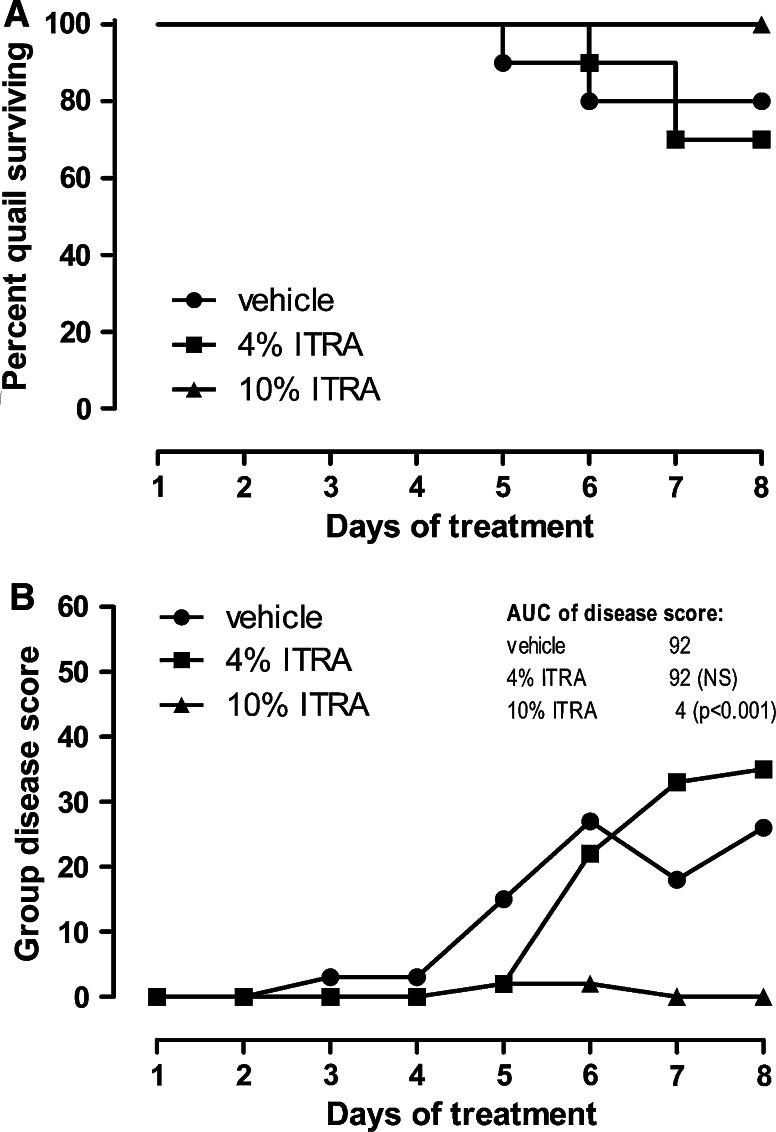
Kaplan–Meier survival curve (**a**) and cumulative disease score (**b**) of groups of quails inoculated intratracheally with 5 × 10^6^ spores of *A. fumigatus*, treated with nebulized ITRA nanosuspension at a concentration of 4 or 10 % (*n* = 10/group). A cumulative disease score was generated by adding up all scores of all animals on the respective day. For statistical analysis of the resulting group disease score, the area under the score curve (AUC) was calculated and compared using the Chi-square test with α-adjustment for multiple comparisons (inserted table)

In quails inoculated with the lower dose of 5 × 10^6^ conidia, the disease development in the vehicle-treated group was comparable to the disease development in the pilot experiment. On day 8, 2 animals had died and 3 others had obvious symptoms of disease, which were pronounced in one of the 3 animals. Gross pathology of animals which had died revealed unspecific signs of multiple organ failure including lung congestion, reddish discoloration of the kidneys, and liver discoloration and swelling. No specific signs of fungal infections of the lungs or air sacs were seen. In animals which had survived until day 8, either no findings or minimal findings presenting as few discolored spots in the lung parenchyma were recorded. Air sacs were free of fungal deposits or granulomas. Once daily treatment with nebulized ITRA nanosuspension for 30 min at a concentration of 10 % resulted in a complete protection of the animals from aspergillosis development. Gross pathology on day 8 of all animals of the treatment group revealed normal air sac membranes and lungs. Neither fungal deposits or granulomas nor treatment-related changes were seen, paralleling the findings in the vehicle-treated animals. With the exception of one animal, which experience equivocal signs of disease on days 5 and 6 only, no other symptoms were recorded. While in the treated group all animals survived, the log-rank test failed to show significance, since in the vehicle group only 2 out of 10 animals had died. However, on the level of the disease score, the treatment effect was significant (*p* *<* 0.001). The quails exposed to 10 % ITRA gained more weight as the quails exposed to vehicle or 4 % ITRA (data not shown). In the 4 % ITRA group, a tendency toward retarded development of symptoms and lethality was observed, but on day 7 following termination of treatment two affected quails died resulting in an even higher lethality in this group. The disease score paralleled the survival curve, and both, the survival and the disease score development, were not significantly different from the control group (Fig. 2a, b).

In quails inoculated with the high dose of 5 × 10^7^ conidia, the disease development was more rapid and more pronounced as compared to the low-conidia control group. First disease symptoms were seen as early as on day 2 following inoculation. A first lethality was seen on day 3, and by day 5, 50 % of animals had died. No further lethality was observed thereafter. The disease score again paralleled the survival curve reaching its maximum on day 6. Gross pathology of animals which had died revealed again unspecific signs of multiple organ failure. No specific signs of fungal infections of the lungs or air sacs were seen. In animals which had survived until day 8, again either no findings or minimal findings representing as few discolored spots in the lung parenchyma were made. Air sacs were free of fungal deposits or granulomas.

**Fig. 3:**
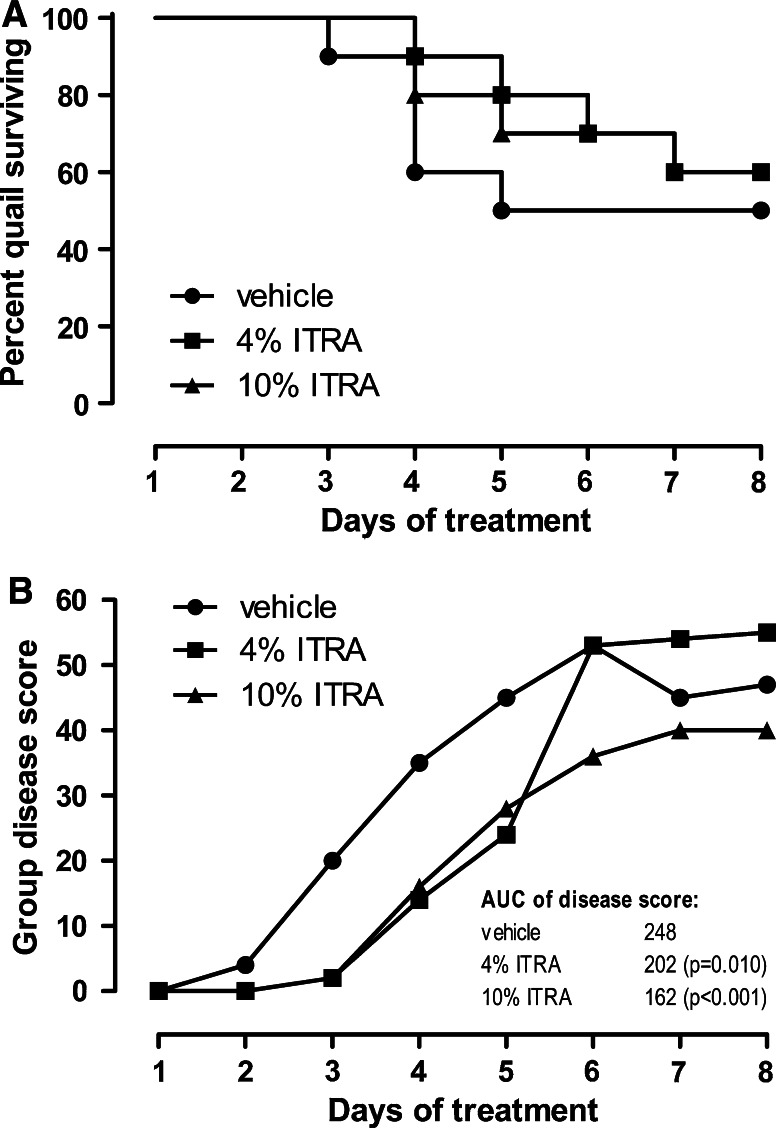
Kaplan–Meier survival curve (**a**) and cumulative disease score (**b**) of groups of quails inoculated intratracheally with 5 × 10^7^ spores of *A. fumigatus* (*n* = 10/group). A cumulative disease score was generated by adding up all scores of all animals on the respective day. For statistical analysis of the resulting group disease score, the area under the score curve (AUC) was calculated and compared using the Chi-square test with α-adjustment for multiple comparisons (inserted table)

The treatment with ITRA nanosuspension (4 and 10 % suspension) resulted in a retarded disease development. While the treatment effect was found to be highly significant for the disease score development for both treatment groups, the improvement in survival was small and did not reach the level of significance. With both treatments, no complete protection from lethality was obtained. No clear dose response for the survival could be observed, but there was a tendency that the disease score was lower in the 10 % group as compared to the 4 % group (Fig. 3a, b). Gross pathology of animals which had died and in those which had survived until day 8 was not different from vehicle-treated animals.

## Discussion

Pulmonary aspergillosis is caused by inhalation of spores of *Aspergillu*s species. A clinically manifest infection occurs only under certain circumstances, such as a very high exposure with spores, or if the bird has an impaired immune system, i.e., if the innate defense mechanisms do not succeed in eliminating the infection [18]. While in rodent models of pulmonary aspergillosis, an immune suppression is required to enable pulmonary infection [16], juvenile but not adult pigeons, and juvenile quails can be infected in the absence of systemic immune suppression [5, 24]. For the present study, we selected 10-day-old quails as model species, since it had been shown previously, that in quails of this age, the intratracheal instillation of *A. fumigatus* spore suspension induced a pulmonary aspergillosis with high pulmonary burden of *A. fumigatus*, leading to clinically manifest disease and mortality within up to 10 days, with subsequent complete recovery in surviving animals [24]. While pulmonary aspergillosis was verified by measuring colony-forming units per gram lung parenchyma, fungal granulomas were seen only in two out of 60 quails inoculated. Pathological findings were in line with systemic mycosis as primary cause of death, and systemic voriconazole treatment, while resulting in increased survival, had little effect on the pulmonary pathology in this study [24]. In our pre-study, we could verify that an intratracheal inoculation of 5 × 10^6^ *A. fumigatus* spores is adequate to induce a clinically manifest disease in >50 % of inoculated quails, resulting in modest mortality. An inoculation with a tenfold higher dose of spores resulted in a more rapid development of the disease symptoms, but again not all animals inoculated developed active disease (Fig. 1). Gross pathology failed to demonstrate lung selective aspergillosis, and no fungal lawn or granulomas were induced, as reported previously for this model [24]. It was decided to evaluate the treatment effect of inhaled ITRA in quails inoculated with both 5 × 10^6^ and 5 × 10^7^ spores per animal. The mortality rate in the 5 × 10^6^ spores group was comparable to previously published data [24].

Although inhalational administration is well established as a treatment option for humans, little is known about this application form in birds. Nebulization of drugs is occasionally used for vaccination or for drug administration [9, 17], but data on treatment of pulmonary aspergillosis using the inhalation route are limited to few case studies [7]. We have previously shown that a stable nanosuspension of ITRA can be generated and successfully nebulized with pressurized air nebulizers [20]. Single and repeated inhalations in rats and quails were well tolerated and resulted in high and persistent lung tissue concentrations, while the plasma exposure was very low [20, 21]. In gray parrots and falcons, the inhalation procedure could be conducted without inducing stress by covering a small cage with a transparent tight cover and nebulizing this chamber (Rundfeldt et al., unpublished observation). This opened up the option that ITRA inhalation could be a targeted treatment option of lung aspergillosis, preventing the systemic toxicity of azole derivatives, which normally requires a careful balancing of dose and adverse effects [3], while at the same time omitting the stress associated with oral treatment. In the present study, we could show that once daily inhalation of a nebulized 10 % nanosuspension of ITRA could completely block the development of aspergillosis in quails following inoculation of 5 × 10^6^ *A. fumigatus* spores, and this treatment regimen was well tolerated. No treatment-related symptoms were observed, and the dissection of the surviving animals on day 8 revealed no macroscopic abnormalities. The pharmacological activity of the 10 % suspension is comparable to results obtained following once daily oral administration of 40 mg/kg voriconazole in the same model, while the activity of 20 mg/kg voriconazole had little pharmacological effect, comparable to the inhalation of 4 % ITRA nanosuspension. However, a dose of 20 mg/kg voriconazole administered once daily was shown to result in mild histological as well as macroscopic liver abnormalities in pigeons [3], indicating that a dose of 40 mg/kg is already in the toxic dose range. If the spore inoculation was increased to 5 × 10^6^ spores, both the 4 and 10 % suspension was only capable of retarding the disease development, but the lethality could not be prevented. Our pharmacokinetic studies in quails following inhalation of 1 and 10 % indicated that repeated inhalation of the 10 % suspension resulted in an ITRA lung tissue concentration of well above 50 µg/g lung tissue which was maintained for more than 72 h following the last dose. This tissue concentration is more than 100-fold above the MIC of ITRA for *A. fumigatus* of about 0.5 µg/ml [6]. From these data, we had expected that the 4 % inhalation would have been similarly effective as compared to the 10 % inhalation. However, this was not the case. A possible explanation may be that the inhaled ITRA nanoparticles are dissolving very slowly on the lung surfaces. Crystalline ITRA, even if sized at 250 nm, cannot be pharmacologically active, but only the fraction dissolved can contribute to the suppression of growth of *A. fumigatus*. One possibility to estimate the dissolved fraction is the measurement of the primary active metabolite, hydroxyl-itraconazole (OH-ITRA). Following inhaled administration of 10 % ITRA nanosuspension for 5 days, the tissue concentration of OH-ITRA was found to be in the range of 1 µg/g lung tissue, and this concentration was maintained for 72 h following the last dose [21]. These data indicate that ITRA nanoparticles accumulate in lung tissue and are slowly dissolved to be metabolized in the lung tissue. A reduction in the concentration of ITRA nanosuspension from 10 to 4 % results in reduced deposition of ITRA and hence in reduced availability for dissolution and metabolization to form OH-ITRA. In fact, following single-dose inhalation of a 1 % suspension, the lung tissue concentration of OH-ITRA did by far not reach the MIC of *A. fumigatus* of 0.5 µg/ml [21].

From these data, we can conclude that once daily 30-min inhalation of a 10 % ITRA nanosuspension is required to obtain active tissue concentrations of ITRA and OH-ITRA for the treatment of pulmonary aspergillosis in birds. Having to administer the 10 % suspension, the advantage of local treatment of the lungs without exposing the systemic circulation to ITRA is not achieved, since at this concentration circulating plasma levels of both ITRA and OH-ITRA were found [21]. Nevertheless, the model of intratracheal inoculation of a high load of *A. fumigatus* spores is rather a model of acute than of chronic aspergillosis. In fact, no granuloma formation on air sac membranes and no fungal deposits on the serosae were visible, indicating that the course of the disease is rather acute, presumably with systemic aspergillosis contributing to the unspecific signs of death observed in our study. A hematological spread of pulmonary aspergillosis has been described previously [11, 14]. In chronic noninvasive pulmonary aspergillosis, a lower drug concentration may be also active. However, this can be only evaluated in a clinical setting in affected birds. Studies in gray parrots and in falcons will have to be conducted to evaluate whether a 10 % or a 4 % suspension can be safely inhaled in these species and whether manifest aspergillosis can be successfully treated.
